# Development of a cloud point extraction method combined with ETAAS and ICP-MS for the preconcentration and quantification of silver as nanoparticles in saline samples

**DOI:** 10.1007/s00604-025-07330-7

**Published:** 2025-07-02

**Authors:** María Carmen Barciela-Alonso, Elena Peña-Vázquez, Juan José López-Mayán, Oscar Rodríguez-Arnoso, Pilar Bermejo-Barrera

**Affiliations:** Trace Element, Spectroscopy and Speciation Group (GETEE), Faculty of Chemistry, Instituto de Materiais (iMATUS), University of Santiago de Compostela, Av. das Ciencias, s/n 15782, Santiago de Compostela, Spain

**Keywords:** Cloud point extraction, Silver nanoparticles, ETAAS, SP-ICP-MS, Seawater analysis

## Abstract

The increasing industrial use of silver nanoparticles (AgNPs) has led to interest in their determination in different types of environmental complex samples, including saline matrices such as aquaculture waters. A simple, reliable, and green analytical method using instrumentation available in most of the laboratories has been developed for the determination of silver as nanoparticles in saline samples. Cloud point extraction (CPE) was used for the separation of AgNPs from seawater, and silver was then determined in the surfactant-rich extracts by electrothermal atomic absorption spectrometry (ETAAS). Parameters related to ETAAS and the cloud point extraction procedure (Triton X-114 concentration, volume of complexing agent (EDTA), pH and type of buffer, effect of repetitive extraction, matrix modifier, etc) were evaluated. A concentration of Triton X-114 of 12% (v/v), a volume of 750 μL of saturated EDTA, and a pH of 7.0 were selected as the optimum conditions for the separation of AgNPs. Standard addition calibration solutions in a concentration range from 0 to 3.0 μg L^−1^ of AgNPs were subjected to the CPE procedure and used for silver determination by ETAAS. The LOD and LOQ were 0.038 and 0.125 μg L^−1^ of silver (as AgNPs), respectively. The method is precise (whole procedure 9.7%) and accurate (recovery percentages 99±4%), showing a good selectivity for the extraction of AgNPs, while ionic Ag remains in the aqueous phase. Moreover, the results of size analysis of the nanoparticles in the surfactant extracts of seawater samples spiked with AgNPs (40 and 60 nm) by SP-ICP-MS and TEM were in good agreement.

**Graphical Abstract:**

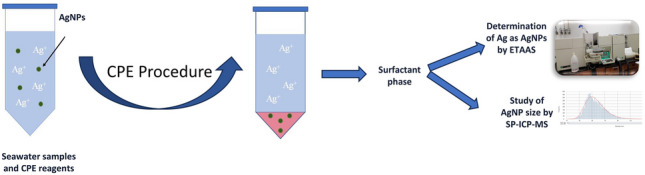

**Supplementary Information:**

The online version contains supplementary material available at 10.1007/s00604-025-07330-7.

## Introduction

In recent years, the use of nanomaterials has increased in different industrial sectors due to their specific properties because of their small size. Inorganic nanoparticles are a type of nanomaterial with many applications in different areas. According to the Nanotechnology Product Database (StatNano), silver nanoparticles (AgNPs) and titanium dioxide nanoparticles (TiO_2_NPs) are the most widely used along with silicon oxide nanoparticles [1]. This database includes 1093 products containing AgNPs, most of them in sectors such as medicine, textiles, cosmetics, environment, and home appliances. Some of their applications are due to silver’s antibacterial and fungicidal properties [2–5]. Because of the increasing use of these nanoparticles, they are released into the environment (e.g., water and soil), reaching sewage treatment plants, rivers, and seawater. Nanoparticles present in aquatic systems can be transformed into other species [2, 6], accumulate in marine organisms, be transferred through the trophic chain, and finally reach humans. Several studies have been carried out to evaluate AgNPs bioaccumulation and toxicity in marine organisms. Thus, Wang et al. [7] studied the toxicity, bioaccumulation, and biotransformation of citrate and polyvinylpyrrolidine (PVP) coated AgNPs in marine organisms via marine sediment exposure. The results obtained from 7-day toxicity tests indicated that these NPs did not exhibit toxicity to the amphipod (*Ampelisca abdita*) and mysid (*Americamysis bahia*) at ≤75 mg Kg^−1^ dry wt. However, a 28-day study showed Ag accumulation in the marine polychaete *Nereis virens* exposed to citrate-AgNPs, PVP-AgNPs, as well as AgNO_3_. Authors concluded that AgNPs surface capping agents influenced Ag uptake, biotransformation, and/or excretion. López Mayán et al. [8] studied the bioaccumulation of PVP-AgNPs in *Palmaria palmata* and *Ulva sp.* seaweed. A bioaccumulation study was conducted with seaweed exposed to 0.1 and 1.0 mg L^−1^ PVP-AgNPs (15 nm) for 28 days. Authors concluded that the bioaccumulation changed with the type of seaweed and was not proportional to the concentration. Araújo et al. [9] studied the effects of AgNPs on the metabolism of turbot, *Scophthalmus maximus*, from the proteomic modifications caused by exposure to sublethal concentrations of the nanomaterial.

Several analytical techniques have been used for the determination of the metallic content in inorganic nanoparticles in different matrices [10]. Among the most used techniques are atomic spectrometry or inductively coupled plasma mass spectrometry (ICP-MS), after subjecting the sample to a previous separation step such as cloud point extraction (CPE), capillary electrophoresis (CE), or field flow fractionation (FFF) [11–13]. On the other hand, single particle ICP-MS (SP-ICP-MS) can provide information on the size and concentration of nanoparticles without prior separation [6, 14]. The advantage of ICP-MS over other techniques is its high sensitivity, but the main drawback is the high cost of the instrumentation, which makes it unaffordable for most of the routine analysis laboratories. On the other hand, a major problem when analyzing nanoparticles in seawater samples is the high salt content, leading to interferences during the analysis. One way to avoid this problem is to dilute the sample, with the risk of the concentration level being below the detection limit of the technique. A review about the current trends and challenges in the analysis of the engineered NPs in seawater was reported by Timerbaev et al. [15].

Atomic absorption spectrometry with electrothermal atomization is a more cost-effective technique that is available in most laboratories. Several studies are reported in the literature for the determination of inorganic nanoparticles in different types of samples using CPE and ETAAS. Therefore, Hartmann et al. [16] developed a method for trace determination of AgNPs in water samples (sewage treatment plant and river water). Lopez-García et al. [17] reported a study for speciation of AgNPs and Ag(I) using CPE and ETAAS applied to spiked tap water, mineral water, and two seawater samples. López Mayán et al. evaluated a method using CPE for the preconcentration of AgNPs in wastewater samples by ETAAS [18].

The aim of this work was to develop a sensitive, low-cost, and selective method for the determination of AgNPs in seawater samples using CPE and ETAAS. The method was evaluated for different nanoparticle sizes. The influence of several parameters affecting CPE and ETAAS applied to seawater samples is described in detail. In addition, the SP-ICP-MS technique was used to check for variations in nanoparticle size during the extraction process.

## Materials and methods

### Instrumentation

An atomic absorption spectrometer 1100B model equipped with a HGA 700 graphite furnace, deuterium background correction, and an AS70 autosampler (Perkin Elmer, USA) was used for Ag determination in the CPE extracts. A NexION^®^ 2000 instrument (PerkinElmer, USA) operating in SP-ICP-MS was used to obtain AgNPs concentration and size distribution. More details about the instrumentation are provided in the Supplementary information.

### Reagents

All the details about reagents are also provided in the Supplementary information.

### Cloud point extraction procedure

Cloud point extraction procedure was used for AgNPs extraction. Therefore, a volume of 40 mL of seawater sample was introduced in a conical tube. Then, 750 µL of saturated EDTA, 500 µL of 12% v/v Triton X-114, and 500 µL of buffer solution pH 7.0 (1.25 M acetic acid/1M acetate) were added. The suspension was vortexed and then introduced in a water bath at 60 °C for 30 min. The tubes were then centrifuged at 4500 rpm at 4 °C for 15 min. The supernatant phase was discarded, and the surfactant phase was used for analysis by ETAAS and SP-ICP-MS. The determination of silver in the surfactant phase by ETAAS requires a digestion of the extract before the analysis. Ultrasonic-assisted acid digestion was carried in an ultrasonic bath at 60 °C for 30 min after the addition of 25 µL of 69% (w/w) HNO_3_ to the sample extracts.

In the experiments by SP-ICP-MS, the surfactant phase was diluted with ultrapure water 200 times before the analysis.

### Silver determination by ETAAS

Silver determination in the digested CPE surfactant-rich phase was performed by ETAAS. The analysis was carried out using a hollow cathode lamp working at 15 mA, with a wavelength of 328.1 nm and a spectral bandwidth of 0.7 nm. A deuterium background correction lamp and an integration time of 3 s were used. The temperature program of ETAAS is shown in Table 1.

**Table 1:** Temperature program of ETAAS for Ag determination in the CPE extracts

Step	Temperature (ºC)	Ramp time (s)	Hold time (s)	Argon Flow (mL min^−1^)
Dry	150	25	25	300
Mineralization	1300	20	20	300
Atomization	1800	0	5	0(read)
Cleaning	2500	3	3	300

A mixture of palladium nitrate and ascorbic acid in concentrations of 50 mg L^−1^ and 172 mg L^−1^, respectively, was used as a chemical modifier. The sample volume injected was 20 µL.

### AgNPs analysis in the CPE extracts by SP-ICP-MS

CPE extracts were analyzed by SP-ICP-MS to evaluate possible changes in nanoparticle size during the extraction process. CPE extracts were diluted with ultrapure water before the analysis by SP-ICP-MS using the operational conditions shown in Table 2. Syngistix™ Nano Application software was used for data treatment in the analysis by SP-ICP-MS. The data treatment using this software requires measurement of the sample flow rate and transport efficiency. The transport efficiency was estimated using PEG-COOH gold nanospheres of nominal diameter of 50 nm from Perkin Elmer (with a diameter measured by TEM of 51 nm). A suspension of 1.016 × 10^5^ part mL^−1^ was introduced in the ICP-MS at a sample uptake rate of 0.21 mL min^−1^, and ^197^Au isotope was monitored. An external Ag ionic calibration between 0 and 2.0 µg L^−1^ (0, 0.5, 1.0, and 2.0 µg L^−1^) prepared in ultrapure water was used for SP-ICP-MS analysis. The AgNPs concentration and size distributions in the diluted extracts were directly obtained from the software after analysis.

**Table 2:** Operational conditions for AgNPs analysis by SP-ICP-MS

Parameter/Component	Type/Value/Mode
Nebulizer	CT R^+^ Meinhard
Spray chamber	5 ºC refrigerated glass cyclone chamber with Peltier PC^3X^
Cone material	Nickel
Radiofrequency power	1600 W
Plasma gas flow	15.0 L min^−1^
Auxiliary gas flow	1.2 L min^−1^
Nebulizer gas flow	1.15 L min^−1^
Sample uptake rate	0.21 mL min^−1^
Dwell time	50 µs
Acquisition time	100 s
Readings	2**×**10^6^
Operation mode	Standard
Isotope	^107^Ag
Rejection parameter q (RPq)	0.25
Transport efficiency, TE(%)	11%

### Statistical analysis

Syngistix™ Nano Application software (Perkin Elmer) was used for data treatment in the analysis by SP-ICP-MS, while Statgraphics XVIII (Warrenton, USA) and Microsoft Excel were used for the statistical analysis of the data obtained by ETAAS in this research work.

## Results and discussion

ETAAS is an inexpensive analytical technique for the determination of the total elemental content, and prior separation is necessary to selectively determine the silver content in the form of nanoparticles. Seawater samples have a very complex matrix due to their high salinity, causing interferences in analytical methods. The removal of the matrix (mostly NaCl) is important to decrease interferences and background signal [19]. In addition, the concentration levels of nanoparticles in the samples are very low, so pre-concentration steps are necessary. The CPE procedure allows the separation of the nanoparticles from the saline matrix, separating the ionic form from the nanoparticulate at the same time as the pre-concentration of the analyte takes place. Another advantage of CPE is that it uses small volumes of reagents, which are non-toxic, and the cost is low. The main drawback is that the technique is time-consuming in comparison to direct analysis using, e.g., SP-ICP-MS. However, in this last technique, the accurate analysis of the size and concentration of nanoparticles will also depend on the amount of dissolved silver present in the sample.

Experiments were carried out to select experimental conditions for selective AgNPs extraction from saline matrices using CPE, as well as the optimum conditions for the analysis of Ag in the surfactant-rich phase from CPE by ETAAS.

### Optimization of the operational conditions for Ag determination by ETAAS

Electrothermal atomic absorption spectrometry was used for Ag determination in the surfactant phase from CPE. This technique was selected because of its low cost of analysis as well as its availability in most chemical analysis laboratories. A study was performed to select the conditions for the different steps of the temperature program as well as the selection of matrix modifiers.

The sample matrix can affect both the extraction process and the determination by ETAAS. The mixture of Pd nitrate-Mg nitrate had been used as a chemical modifier to minimize matrix interferences in the analysis of Ag in the surfactant-rich phases obtained after CPE of AgNPs in a previous study applied to wastewaters [18]. However, this mixture was not able to reduce the background signal from the extracts of seawater homogenized with ethanol, and poor precision was obtained in the analytical signals, even if two drying and two mineralization steps were included in the graphite furnace program.

Finally, after several experiments, it was decided to select a mixture of Pd nitrate and ascorbic acid at concentrations of 50 mg L^−1^ and 172 mg L^−1^, respectively, as matrix modifier. Bermejo-Barrera et al. [19] critically studied the use of chemical modifiers for the quantification of high, medium volatility and refractory metals in seawater. The background absorbance depended on the salt content in the sample, mineralization and atomization temperatures, and the chemical modifier used. For Ag (medium volatility), the background was higher than for refractory elements, and the use of reduced palladium (e.g., palladium nitrate + ascorbic acid) improved the analytical characteristics such as sensitivity and reduced the interferences of sodium chloride and potassium sulfate.

To optimize the ETAAS temperature program, a 10-fold diluted CPE seawater extract digested with nitric acid was spiked with 5 µg L^−1^ of AgNPs of 20 nm. The results obtained, absorbance signal and background signal, for the optimization of mineralization and atomization temperatures are shown in Figure S1 (Supplementary information). Considering the absorbance signals of the analyte and background, as well as the peak shape, 1300 °C and 1800 °C were selected as the optimum temperatures for mineralization and atomization, respectively.

### Optimization of the cloud point extraction procedure

After selecting the ETAAS measurement conditions, the parameters affecting the CPE process were evaluated. The effect of the volume of saturated EDTA, concentration of Triton X-114, and pH in the AgNPs extraction were studied.

#### Optimization of the volume of chelating agent (saturated EDTA)

The first parameter studied was the volume of saturated EDTA added. EDTA is added to the sample to complex ionic silver to prevent its extraction to the surfactant phase. The starting conditions used for the optimization of the CPE procedure were those used in our previous work [18], with some modifications.

Therefore, a volume of 40 mL of seawater sample spiked with 5 µg L^−1^ of AgNPs (20 nm) was introduced in a conical tube. Volumes of 0.5 mL of 12% Triton X-114, 0.4 mL sodium acetate (1 M), 0.1 mL of acetic acid (1.25 M), and variable volumes of saturated EDTA (0.5, 0.75, 1.0, 1.25, and 1.5 mL) were then added. The samples were vortexed and introduced in a water bath at 60 °C for 30 min. After this time, the samples were centrifuged at 4500 rpm for 20 min. The supernatant phase was discarded, and the surfactant phase, containing the AgNPs, was used for the analysis by ETAAS after ultrasound-assisted digestion with nitric acid. The experiment was carried out in triplicate for each volume of EDTA and blank. The digested extract was diluted ten times for the analysis by ETAAS using the measurement conditions described above.

**Fig. 1:**
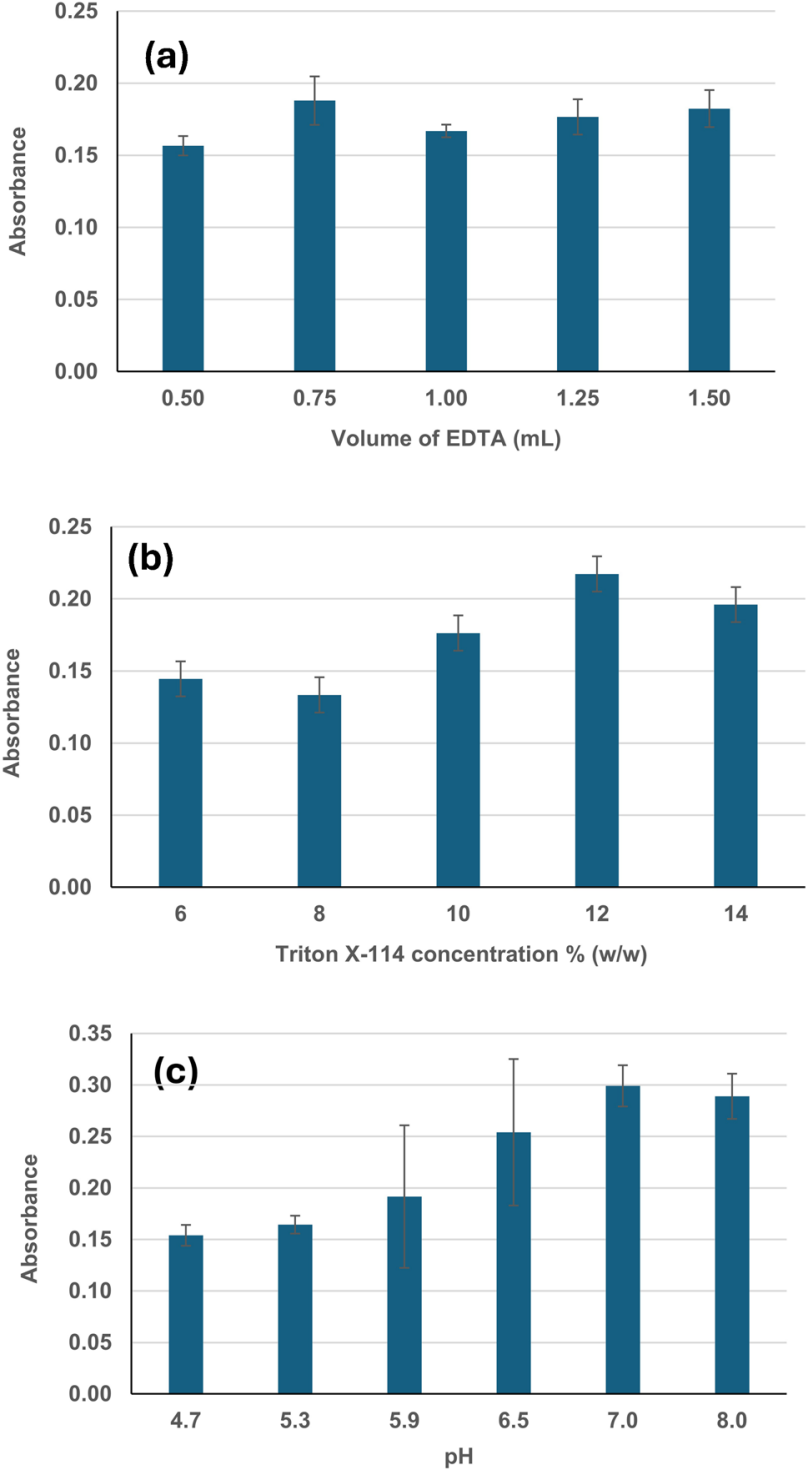
Influence of (**a**) the volume of saturated EDTA added in the AgNPs extraction process, (**b**) concentration of Triton X-114, (**c**) pH

The results obtained are shown in Fig. 1a. The one-way ANOVA test performed using the software Statgraphics XVIII (Warrenton, USA) showed *P*-values lower than 0.05; then, this confirms statistically significant differences between the average absorbances obtained for the EDTA volumes studied at a 95% significance level. Based on the results obtained, a volume of saturated EDTA of 750 µL was selected.

#### Optimization of the concentration of Triton X-114

The second parameter evaluated was the percentage of the surfactant Triton X-114. The study was performed by preparing suspensions of AgNPs in seawater, as in the EDTA effect study, and adding 750 µL of saturated EDTA, the same volumes of acetic acid and sodium acetate, as well as 0.5 mL of Triton X-114 of different concentrations (6, 8, 10, 12, and 14% (w/w)). Triplicates of each concentration level were used with their corresponding blanks. CPE was performed, and diluted extracts were analyzed. Absorbances are shown in Fig. 1b. The best results were obtained for 12% (w/w) Triton X-114; therefore, this concentration was selected for further experiments.

The optimum concentration was higher than that used in other studies. López-Mayan et al. [8] used an optimum concentration of 8.6% (w/w) Triton X-114 in the analysis of wastewaters, while Wimmer et al. [6] employed 10% (w/w) Triton X-114 to obtain seawater extracts before the measurements by ICP-MS.

#### Optimization of pH

The last parameter evaluated was the pH of the medium. For this, the CPE process was performed, adding the previously selected volume of EDTA and concentration of Triton X-114 and varying the pH in a range from 4.7 to 8 (4.7, 5.3, 5.9, 6.5, 7.0, 8.0) using the buffer solution of acetic acid (1.25 M) and sodium acetate (1M). As in the previous cases, the experiment was carried out in triplicate for each pH value, as well as the corresponding blanks. After analysis of the results obtained (Fig. 1c), pH = 7 was selected for the extraction of AgNPs from seawater by CPE, similarly to a previous study in our research group using fresh waters [8].

It was observed that the absorbances increased and stabilized at pH 7.0 in the acetic acid/acetate buffer. Another experiment was carried out at pH 9.0 and 10.0 using a phosphate buffer, and obtaining the maximum signal at pH 10.0. However, in this case, the extraction of AgNPs vs. ionic silver was not selective, probably due to the precipitation of silver as phosphate.

#### Use of a double cloud point extraction procedure

Repetitive CPE procedures have been proposed to concentrate nanoparticles before transmission electron microscopy combined with energy dispersive X-ray spectrometry (TEM-EDX) [20]. As the sample analyzed in the present study is seawater, which contains a high concentration of salts, experiments were carried out using a double CPE procedure to reduce the background signal in the ETAAS determination. The experiment was carried out by performing a first CPE and separating the surfactant phase, which contains the AgNPs. This organic rich phase was then weighed, and 40 mL of ultrapure water and the reagents necessary to perform the second extraction (EDTA, Triton X-114, and sodium acetate/acetic acid buffer) were added. After centrifugation, both phases were separated, and the surfactant phase was digested with HNO_3_ before ETAAS analysis. In the analysis of the aqueous phase, absorbance values were similar, indicating that there was no loss of AgNPs in the double extraction. After analysis of Ag in the surfactant phase, it was observed that although there was a reduction in the background signal, there was no improvement in the precision of the method. At high concentrations of nanoparticles, RSD % values higher than 10% were obtained (20% and 28% for AgNPs concentrations of 1.0 and 1.5 µg L^−1^, respectively). Considering these results, it was decided to discard the double extraction procedure. The precision in the measurements was improved with the addition of 25 µL of 69%(w/w) HNO_3_ to the extracts instead of ethanol [8] and sonicating for digestion and homogenization before ETAAS analysis.

The final optimized CPE procedure is described in the “Cloud point extraction procedure” section.

### Analytical figures of merits

#### Calibration. Influence of particle size

In our previous study, for the determination of AgNPs in wastewater samples using CPE and ETAAS [18], two different calibration methods were compared: the standard addition method using surfactant extracts to prepare AgNPs standards and a standard addition subjected to the CPE procedure. Statistically significant differences were observed between the slopes of both calibrations; therefore, standard addition subjected to the CPE procedure was selected to minimize the effect of the interferences due to the matrix of the sample. Considering the results of our previous study and the fact that the seawater sample matrix is much more complex due to the high salt content, it was decided to use the standard addition method subjected to the CPE process.

To perform the calibration, aliquots of seawater spiked with concentrations of AgNPs of 0, 0.5, 1.0, and 2.0 µg L^−1^ were subjected to the CPE procedure. The procedure was carried out in duplicate for each concentration. The surfactant phase was then separated and analyzed in triplicate by ETAAS. To assess whether the size of the nanoparticles influenced the sensitivity of the method, calibration was performed using three different sizes of AgNPs (20, 40, and 60 nm covered with citrate). The calibration equations (absorbance vs. Ag concentration) obtained were *A* = 0.0596 [Ag] + 0.009, *r* = 0.998 (for 20 nm), *A* = 0.0607 [Ag] + 0.00092, *r* = 1.000 (40 nm), and *A* = 0.0623 [Ag] + 0.0148, *r* = 0.995 (60 nm).

The slopes of these calibrations were compared using a t-test, and no significant differences were observed at a 95% significance level. This means that the sensitivity of the method does not depend on the size of the nanoparticle used to perform the extraction process.

#### Sensitivity

The sensitivity of the method was studied by the calculation of the limit of detection (LOD) and quantification (LOQ). These limits were calculated according to IUPAC recommendations [21], considering the standard deviation of the measurements of a blank (subjected to the CPE procedure and digested with nitric acid in the US bath) and the slope of the analytical calibration curve obtained. The LOD (3**×** blank standard deviation/slope) and LOQ (10 **×** blank standard deviation/slope) obtained were 0.038 µg L^−1^ and 0.125 µg L^−1^, respectively.

Table 3 shows the characteristics of the methods used for the analysis of AgNPs in environmental waters after CPE. As it can be observed, the studies focusing on [6] or providing some information on AgNP_S_ in seawater [17, 22, 23] are very scarce. The calibration ranges and the detectors used were also very different: ICP-MS and SP-ICP-MS [6], ETAAS with high resolution continuum source (HRCSAAS) [17], total reflection X-ray fluorescence spectrometry (TXRF) [22], and colorimetry [23].

**Table 3:** Methods for analysis of AgNPs in environmental waters after cloud point extraction

Authors	Ref.	Type of water	Type of AgNPs	Detection	Calibration range	LOD	Recovery	Precision (RSD %)
Wimmer et al.	[6]	Wastewaters, matrices with increasing salinity and **seawater**	Citrate coated; AgCl-NPs, Ag_2_S-NPs	ICP-MS SP-ICP-MS	5–200 ng L^−1^	0.2 ng L^-1^; 8 nm (in size)	88–94 %	-
Yang et al.	[11]	Wastewater, river and lake water	Citrate coated	ICP-MS	0.16–4 µg L^−1^	5 ng L^−1^	79–123 %	8%
Hartmann et al.	[16]	River water, treated and untreated wastewater, synthetic solutions	Citrate coated	ETAAS (Zeeman correction)	1.25–50 ng L^−1^	0.7 ng L^−1^	> 80 %	1.8–12.8 %
López-García et al.	[17]	Tap, bottled, mineral and two **seawater** samples	Citrate coated	ETAAS (HRCSAAS)	0.005–0.1 µg L^−1^ (3 central pixels); 0.2–4.0 µg L^−1^	2 ng L^−1^ (3 central pixels); 0.05 µg L^−1^	96–105 %	4.60%
Lopez-Mayan et al.	[18]	Wastewaters	Citrate coated	ETAAS (deuterium correction)	0–10 µg L^−1^	0.04 µg L^−1^	97–106 %	4.30%
Bahadir et al.	[22]	Mineral, tap, river and **seawater**	Citrate coated	TXRF	1–1000 µg L^−1^	0.33 µg L^−1^	82–100% (external calibration); 94% (spiked seawater)	16%
Wu et al.	[23]	Tap water and **seawater**	Citrate coated	Colorimetric	17–425 µg L^−1^	1 µg L^−1^	98–102 %	-
Liu et al.	[24]	River and lake water. WWTP effluents and influents	PVP-AgNPs and commercial AgNPs	ICP-MS	0.1–146 µg L^−1^ (spiked levels)	6 ng L^−1^	57–116% (88.8% for 100 µg L^−1^)	5.60%
Wei et al.	[26]	River, lake and tap water	Ag_2_S NPs	SP-ICP-MS	0.25–5 µg L^−1^	22 nm (size); 5x10^4^ part L^−1^ (concentration)	76–106 % (particle number)	-
Zhou et al.	[30]	River and lake water. WWTP effluent	Ag_2_S-NPs	LC-ICP-MS	0.1–100 µg L^−1^	8 ng L^−1^	81.3–96.6%	< 4.9%
Barciela-Alonso et al.	Present study	**Seawater**	Citrate coated	ETAAS (deuterium correction)	0–3 µg L^−1^	0.038 µg L^−1^	99±4 %	1.7% (repeatib.); 9.7% (whole procedure)

The limits of detection reported in the literature depend mainly on the type of technique used for the detection [15], with the minimum value obtained by Wimmer et al. [6] combining CPE and ICP-MS and diluting the surfactant-rich phase with aqueous ethanol. Liu et al. [24] were the first research group that determined Ag as AgNPs after CPE with Triton X-114, microwave digestion of the CPE extract, and ICP-MS detection, and obtained a LOD of 6 ng L^−1^. The method was applied to river, lake, and wastewater treatment plant (WWTP) waters. In a previous study by Bahadir et al. [22] that used CPE with TXRF detection for the simultaneous determination of silver and gold nanoparticles, obtained a limit of detection in aqueous samples 0.33 µg L^−1^. Analysis of AgNPs using CPE with Triton X-114 and gold nanoparticles stabilized with Tween 20 for colorimetric assay provided a limit of detection of 1 µg L^−1^ in tap water and seawater [23].

The detection limit obtained in this study is better than that reported by López García et al. [17] (0.05 µg L^−1^), performing the calibration in a range of concentrations similar to that of the present work (0.2–4.0 µg L^−1^). However, as these authors use a high-resolution source spectrometer, they can achieve a limit of detection as low as 2.0 ng L^−1^, considering only the three central pixels for analysis and decreasing the calibration range to 0.005–0.1 µg L^−1^. However, this detection limit is somewhat higher than that obtained by Hartmann et al. [16] (0.7 ng L^−1^) for the determination of AgNPs in river water samples and wastewater treatment plant effluents. This difference in sensitivity may be due to the high salt content of seawater and to the different types of instruments or background correction system in the ETAAS equipment used. A Zeeman-corrected atomic absorption spectrometer was used in the study reported by Hartmann et al. [16].

The LOD and LOQ obtained in a previous study of our research group were 0.04 and 0.13 µg L^−1^ using fresh water and a graphite furnace with deuterium background correction, and they were then very similar to those achieved using a seawater matrix. Therefore, it seems that the combination of CPE and ETAAS was not strongly affected by the presence of the saline matrix.

#### Precision

Precision was assessed through repeatability and precision of the whole procedure. The repeatability was evaluated by performing ten repeated ETAAS measurements of a CPE extract obtained from a seawater sample spiked with 1 µg L^−1^ of 20 nm AgNPs diluted ten times before the analysis. The relative standard deviation obtained was 1.7%. The precision of the whole procedure was assessed using a seawater sample spiked with two concentration levels (0.5 and 2 µgL^−1^) of 20 nm AgNPs, subjected in triplicate to the CPE procedure. The average RSD was 9.7%. The relative standard deviation values are less than 10%, so we can conclude that the method presents good precision.

#### Analytical recovery

The lack of certified reference materials is one of the main problems found in the analysis of engineered nanoparticles, specifically in seawater [15]. Since certified reference materials of AgNPs are not available, the accuracy of the method was evaluated by calculating the analytical recovery. The analytical recovery was assessed using a seawater sample spiked with three concentration levels (0.5, 1.0, and 2.0 µg L^−1^) of 20 nm and 40 nm and 60 nm AgNPs subjected to the CPE procedure in triplicate. The mean analytical recoveries obtained were 101±8 %, 90±5%, and 97±6% for spiked AgNPs of 20, 40, and 60 nm, respectively. The recovery percentages obtained are around 100% for the three particle sizes studied, so it can be concluded that the method presents good analytical recovery using the standard addition method. Bahadir et al. [23] also observed by CPE-TXRF that the recovery of AgNPs in spiked seawater was approximately 40–50% instead of 94% if the standard addition method is not applied. Moreover, our study shows that the recovery percentage does not depend on the nanoparticles size.

#### Selectivity

The aim of this work was to develop a selective method for the determination of silver nanoparticles, so that in the extraction process, the ionic silver would remain in the aqueous phase. Experiments were performed to evaluate the capacity of the method to separate AgNPs and ionic silver. Therefore, seawater sample spiked with 2 µg L^−1^ of ionic Ag was subjected to the CPE procedure in triplicate. Silver was then determined in the acid-digested surfactant-rich phase by ETAAS. The extraction efficiency of ionic silver obtained was 4%. These results indicate that the method is selective for the extraction of AgNPs and that ionic silver remains in the supernatant phase.

### Analysis of AgNPs in the extracts by SP-ICP-MS

Single particle inductively coupled plasma mass spectrometry (ICP-MS) was used to determine the size of AgNPs in the extracts after submitting a seawater sample to the CPE procedure. The objective is to check whether a change in particle size occurs during the extraction process.

For the experiment, a seawater sample was spiked with 2 µg L^−1^ of AgNPs. Two different sizes of AgNPs were tested: 40 nm silver nanospheres (size by TEM: 41 ± 5 nm) and 60 nm silver nanospheres (size by TEM: 59 ± 6 nm), both stabilized with citrate. The CPE protocol was applied in triplicate, and the surfactant-rich phase extracts obtained (no digestion with nitric acid) were kept in the fridge for 24 h at 4 °C before the analysis by SP-ICP-MS.

**Fig. 2:**
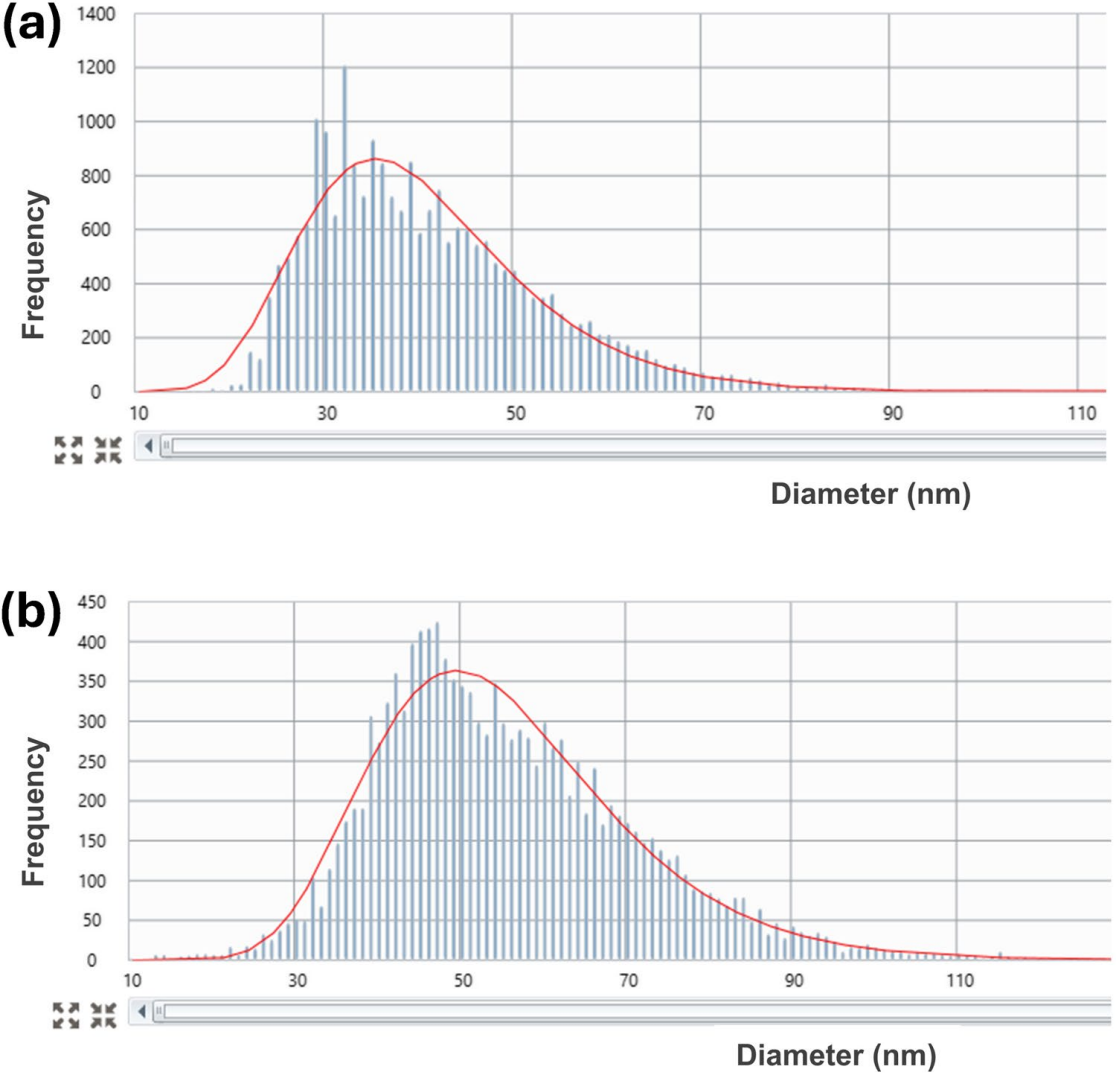
SP-ICP-MS histogram of a CPE extract of a seawater sample spiked with (**a**) 40 nm and (**b**) 60 nm AgNPs

The instrumental conditions applied for the SP-ICP-MS measurements (Table 2) and the instructions for calibration and calculation of transport efficiency are included in the “AgNPs analysis in the CPE extracts by SP-ICP-MS” section. The surfactant-rich phases were diluted 200 times with ultrapure water before the measurements. Each extract was diluted and analyzed in duplicate. The mean values measured showed a good agreement with the diameters obtained by TEM for both particle sizes: 40.0 ± 1.0 nm vs. 41 ± 5 nm (TEM) and 55.2 ± 0.5 nm vs. 59 ± 6 nm (TEM). The relative standard deviations of the mean (*n* = 12) were 3 and 1% for particle sizes of 40 and 60 nm, respectively, indicating a good precision in diameter size measurements. Two examples of size histograms are shown in Fig. 2. The limit of detection in size (LOD_size_) was calculated after the analysis of the rich surfactant extract of a seawater without spiked silver diluted 200 times and using the spreadsheet designed by Laborda et al. [25]. The LOD_size_ obtained was 16.7 nm using the 3σ criteria (3× baseline standard deviation), or 19.8 nm using the 5σ criteria. This LOD is slightly smaller than the value of 22 nm reported by Wei et al. [26] who used CPE and SP-ICP-MS to study the behavior of nano-silver sulfide in environmental waters (lake, river, and tap water). However, Wimmer et al. [6] obtained a LOD_size_ of 6 nm for seawater. The instrumental limit of detection for the concentration of particles (LOD_concentration_) was 5.28 × 10^5^ part L^−1^ using the 5σ criteria to avoid false positives, and referred to sample, it was 1.06 × 10^8^ part L^−1^ (dilution 1:200).

SP-ICP-MS can be a powerful tool to monitor the behavior of AgNPs in waters at concentrations as low as ng L^−1^ [27]. Toncelli et al. [28] had previously investigated the dissolution of AgNPs in seawater microcosm by SP-ICP-MS, observing a higher stability of branched poly(ethyleneimine)-coated AgNPs in comparison to PVP-AgNPs. Wimmer et al. [6] evaluated by CPE and SP-ICP-MS the fate of AgNPs (citrate-coated, AgCl-NPs, Ag_2_S-NPs) in waters with increasing salinity and seawater, and they found that most of them were dissolved after 72 h of incubation. In the present study, the good agreement between the diameters measured by SP-ICP-MS after CPE and the certified values by TEM indicated that the extracts were stable during at least 24 h and that single particle ICP-MS analysis is useful for size analysis in saline matrices as seawater after the cloud extraction procedure.

Cloud point extraction is useful to extract and enrich NPs, but more studies are still needed to understand the mechanisms of the separation of colloidal and dissolved species and model their behavior. Automation of CPE will be another important area of research to improve the efficiency and strengthen the use of this technique in routine analyses [29]. As it can be observed in Table 3, most of the studies in the literature were performed using citrate-coated NPs. The fate of AgCl-NPs and Ag_2_S-NPs was also studied in saline matrices and seawater [6], and Zhou et al. [30] performed the speciation of Ag_2_S and ZnS NPs in lake, river, and wastewaters by liquid chromatography and ICP-MS after CPE. Hartmann et al. [31] also evaluated the influence of nanoparticle coating of AgNPs and matrix components on the CPE. They found that CPE recoveries were between 82 and 105% for all the types of coatings studied (citrate, PVP, 12-mercaptoundecanoic acid, mercaptosuccinic acid, lysine, cysteine, starch, chloride, sulfide, phosphate) except for bovine serum albumin. Moreover, these authors reported the formation of AgNPs after the reduction of ionic silver in the presence of organic matter. Similar studies should be performed with real seawater samples of different salinities, organic matter content, and to model the aging of the NPs in different environmental conditions such as irradiation.

## Conclusion

A simple, selective, inexpensive method for the determination of Ag as nanoparticles in seawater samples has been developed after the optimization of ETAAS and CPE conditions using this saline matrix. Calibration using the standard addition method subjected to the CPE process was necessary to minimize sample matrix effects, and the mixture palladium nitrate-ascorbic acid was used as a matrix modifier for the analysis of the surfactant-rich extract after an easy ultrasound-assisted digestion step with nitric acid. The slopes of the calibration graphs obtained, precision and recovery did not depend on the size of the nanoparticles used. The results obtained with SP-ICP-MS showed that there was no change in the size distribution of the nanoparticles during the CPE process.

Considering the analytical characteristics of the proposed method, and that ETAAS is a low-cost technique available in many analytical laboratories, the developed method could be used for the analysis of AgNPs in seawater samples in environmental analysis laboratories, especially if further developments in extraction automation are achieved in the future.

## Supplementary Information

Below is the link to the electronic supplementary material.

Supplementary file 1 (DOCX 38 KB)

## Data Availability

No datasets were generated or analysed during the current study.
