# Supplementary material for: Development of a cloud point extraction method combined with ETAAS and ICP-MS for the preconcentration and quantification of silver as nanoparticles in saline samples
Source: Mikrochim Acta. 2025 Jul 2;192(8):470. doi: 10.1007/s00604-025-07330-7 (PMC12222420; doi:10.1007/s00604-025-07330-7)

**Supplementary information**

**Development of a cloud point extraction method combined with ETAAS and ICP-MS for the preconcentration and quantification of silver as nanoparticles in saline samples**

**María Carmen Barciela-Alonso***, Elena Peña-Vázquez*, Juan José López-Mayán, Oscar Rodríguez-Arnoso, Pilar Bermejo-Barrera

Trace Element, Spectroscopy and Speciation Group (GETEE), Instituto de Materiais (iMATUS), Faculty of Chemistry, University of Santiago de Compostela, Av. das Ciencias, s/n 15782, Santiago de Compostela, Spain.

Corresponding Authors: María Carmen Barciela-Alonso and Elena Peña-Vázquez

Email corresponding authors: [mcarmen.barciela@usc.es](mailto:mcarmen.barciela@usc.es)

[elenamaria.pena@usc.es](mailto:elenamaria.pena@usc.es)

**Table of contents**

**Instrumentation___________________________________________________Page 3**

**Reagents_________________________________________________________Page 4**

**Fig. 1S___________________________________________________________Page 5**

**Instrumentation**

An atomic absorption spectrometer 1100B model equipped with a HGA 700 graphite furnace, deuterium background correction and an AS70 autosampler (Perkin Elmer, USA) was used for Ag determination in the CPE extracts.

An inductively coupled plasma mass spectrometer (ICP-MS) NexION^®^ 2000 (PerkinElmer, USA) with Kinetic Energy Discrimination (KED) working in single-particle mode (SP-ICP-MS) was used with the Syngistix™ Nano Application 2.5 software (Perkin Elmer) to obtain AgNPs concentration and size distributions.

A thermostatic ultrasonic bath USC 600 TH (VWR, USA), a Consul 22 R centrifuge with rotor RT 280 (Ortoalresa, Spain), a Vortex Reax Top vibrational shaker (Heidolph^TM^, Germany), an analytical precision electronic balance ML204 (Mettler Toledo, Spain) and a pH meter Basic 20 (Crison, Spain) were used to perform the cloud point extraction procedure.

**Reagents**

The silver standard solution (1000 mg L^-1^ in 0.5 M HNO_3_), Triton X-114, sodium acetate anhydride for analysis, sodium hydroxide for analysis, and sodium dihydrogen phosphate monohydrate were obtained from Merck (Germany). Three different sizes of AgNPs from nanoComposix (USA) were used in the experiments: 20 nm Silver Nanospheres, Citrate, NanoXact^TM^ (size by TEM: 20.8 ± 3.0 nm; mass concentration 0.021 g L^-1^), 40 nm Silver Nanospheres, Citrate, NanoXact^TM^ (size by TEM: 41 ± 5 nm; 0.021 g L^‑1^), and 60 nm Silver Nanospheres, Citrate, NanoXact^TM^ (size by TEM: 59 ± 6 nm; 0.020 g L^-1^). The transport efficiency, TE (%), in SP-ICP-MS was measured using 50 nm Gold Nanospheres, PEG-COOH, (diameter by TEM: 51.0 ± 1.9 nm), from Perkin Elmer (USA). EDTA disodium salt dihydrate for analysis, 69% (w/w) nitric acid and 96% (w/v) acetic acid for analysis were provided by Panreac (Spain). Magnesium nitrate (BDH Chemicals Ltd, United Kingdom), palladium nitrate (SCP Science, Canada) and ascorbic acid ≥ 99% (Honeywell Fluka^TM^, USA) were used as chemical modifiers for electrothermal atomic absorption spectroscopy determination.

Other reagents were used to perform the analysis by ICP-MS. They included NexION Setup Solution (1.0 µg L^-1^ of Be, Ce, Fe, In, Li, Mg, Pb, and U in 1% of HNO_3_) from Perkin Elmer; He and Ar, both with a 99.999% of purity, were from Nippon Gases (Spain).

All the solutions were prepared using ultrapure water (18 MΩ cm) obtained from a Milli-Q^®^ purification unit (Millipore Co., USA). All glassware and storage bottles were kept in 10% (v/v) nitric acid for at least 48 h, rinsed three times with ultrapure water, and preserved dried for their use. Disposable plastic labware was used throughout most of the procedures.

**Fig. 1S**. Mineralization (left) and atomization (right) curves for the determination of Ag in the CPE extracts. Analyte signal and background signals are in blue and orange, respectively


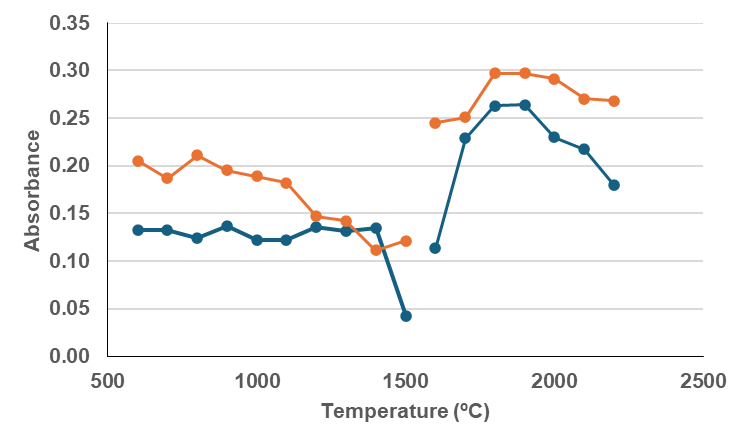

Supplement: Supplementary file 1 — (DOCX 38 KB) [file 604_2025_7330_MOESM1_ESM.docx]
